# Supplementary material for: StrainFLAIR: strain-level profiling of metagenomic samples using variation graphs
Source: PeerJ. 2021 Aug 23;9:e11884. doi: 10.7717/peerj.11884 (PMC8388557; doi:10.7717/peerj.11884)
Supplement: Supplemental Information 1 [file peerj-09-11884-s001.pdf]

S1 SUPPLEMENTARY MATERIALS

S1.1 Third-party tools usage and rational

We propose here a the motivations and precise usage of the third-party tools that are employed in `StrainFLAIR`.

S1.1.1 Graph construction

`vg toolkit` allows to modify the graph including a normalization step. Normalization consists in deleting redundant nodes (nodes containing the same sub-sequence and having the same parent and child nodes), removing edges that do not introduce new paths, and merging nodes separated by only one edge.

For each cluster, if the colored paths of the corresponding graph still describe their respective input sequences, the graph is normalized.

After the concatenation of all computed graphs (one for each cluster), the final single variation graph is indexed using `vg toolkit`. Indexing a graph allows a fast querying of the graph when mapping reads. Indexing uses two file formats: *XG*, which is a succinct graph index which presents a static index of nodes, edges and paths of a variation graph, and *GCSA*, a generalized FM-index to directed acyclic graphs. A *SNARLS* file is also generated, describing snarls (a generalization of the superbubble concept (Paten et al., 2018)) in the variation graph and similarly allowing faster querying.

S1.1.2 Mapping reads

`vg toolkit` offers two sequence-to-graph mappers. The first one, `vg map`, outputs one or several final paths for each alignment. However, in case of several alignments with equal mapping scores, only one is randomly chosen. In order to get more exhaustive and accurate results, `StrainFLAIR` uses `vg mpmap` to map reads on the variation graph.

The mapping results are given in *GAMP* format, then converted into *JSON* format with `vg toolkit`, describing, for each read, the nodes of the graph traversed by the alignment.

S1.2 Gene-level output by `StrainFLAIR`

Here we present the exhaustive description of information provided by `StrainFLAIR` at the gene level (before strain-level computations). For each colored path `StrainFLAIR` provides the following items:

- The corresponding gene identifier.
- For each reference genome, the number of copies of the gene. Since each unique version of a gene is represented once in the graph, whereas it can exist in several copies in the genome (duplicate genes), the counts and abundances computed correspond to the sum of those copies. Keeping track of the number of copies is important to normalize the counts.
- The cluster identifier to which the colored path belongs.
- For unique mapped reads: their raw number and their number normalized by the sequence length (see Section Querying variation graphs in Methods).
- For unique plus multiple mapped reads: their raw number and their number normalized by the sequence length (see Section Querying variation graphs in Methods).
- The mean abundance of the nodes composing the path, as defined in the manuscript.
- The mean abundance without the nodes of the path never covered by a read, as defined in the manuscript.
- The ratio of covered nodes, as defined in the manuscript.

S1.3 Abundance metrics validation

The output of `StrainFLAIR` provides several metrics to estimate the abundance of the genes detected in the sample.

For validation, we used a combination of LASSO (least absolute shrinkage and selection operator) model and linear model on the simulated dataset to estimate the abundances at the strain-level, as the abundance of a gene is a linear combination of the abundances of the strains it belongs to. As such,

we expect no intercept value for those models and have forced the intercept at zero for the following modeling.

First, a LASSO model was used to perform strain selection. The response variable of the model was the presence or absence of the genes according to the selected metric while the strains, described as their genes content (number of copies), were the predictors. Then, a linear model was constructed with the raw selected metric as the response variable, and only the strains selected by the LASSO model as the predictors. The estimate of the strains relative abundance was thus the coefficients of the linear model associated to the strains and transformed into relative values. For each metric, the sum of squared errors between the real relative abundances and the estimated relative abundances from the linear model was computed. The best metric was then defined as the one minimizing this sum of squared errors.

For the mixtures containing *E. coli* K-12 MG1655, the three expected strains were selected and thus detected using LASSO, except for the mixture containing only 1,000 reads of K-12 MG1655 (representing 0.002% of the mixture, hence very negligible). For all the mixtures, the best metric was the mean abundance computed from the node abundances and by taking into account the multiple mapped reads.

For the mixtures containing *E. coli* BL21-DE3, BL21-DE3 being absent from the reference but very close to K-12 MG1655, we expected to get some detection of K-12 in the results. The three expected strains were selected and thus detected using LASSO, except for the mixture containing only 1,000 reads of BL21-DE3 (representing 0.002% of the mixture, hence very negligible). For the mixtures at 200,000, 100,000, and 50,000 reads of BL21-DE3, the best metric was the mean abundance computed from the node abundances without the abundances at zero, and by taking into account the multiple mapped reads. While for the others, the best metric was the mean abundance computed from the node abundances (including the abundances at zero), and by taking into account the multiple mapped reads.

This approach using linear models was particularly appropriate for this situation where the reference variation graph and the sample contained a small number of strains and thus a small number of predictors for the model. However, this can hardly transpose to a whole metagenomic sample with various species and various strains that would lead to too many predictors and probably confusing the heuristics behind the models. This was confirmed by applying the same methodology above on the mock dataset leading to abundances estimation hardly comparable to expected. Compared to `Kraken2` results, the sum of squared errors of our methodology was approximately 6 whereas for the results with the LASSO model it was around 236. Nevertheless, those results highlighted the relevance of (i) using a metric taking into account the multiple mapped reads and not only the unique mapped reads, and (ii) using our metric of abundance based on the node abundances over raw read counts.

S1.4 Performances

Our benchmarks were performed on the GenOuest platform on a machine with 48 Xeon E5-2670 2.30 GHz with 500 GB of memory and 16 CPUs. Time results (Table S1) are the wall-clock times. We provided rough computation time, mainly in the purpose to show that `StrainFLAIR` can be applied on usual datasets.

Dataset	Step	Items processed	Time	Disk used (GB)	Max mem. (GB)
Simulated	Gene prediction	7 genomes	0m20	0	1.2
	Gene clustering	34,011 genes	0m22	0	0.36
	Graph construction	8,596 clusters	2m44	0.04	1.31
	Graph concatenation	8,596 graphs	0m51	0	0.25
	Indexing graph	1 graph	6m23	0.16	4.24
	Mapping reads	350,000 short reads	15m15	0.16	0.99
	JSON conversion	1 GAMP file	3m58	4.2	0.03
	JSON parsing	1 JSON file + 1 GFA file + 1 pickle file	12m44	0	0.55
	Abundance computing	1 Gene abundances table	0m2	0	0.04
Mock	Gene prediction	91 genomes	1m43	1.02	6.7
	Gene clustering	280,174 genes	3m38	0.14	0.98
	Graph construction	270,712 clusters	41m54	1.12	9.1
	Graph concatenation	270,712 graphs	14m38	0	1.05
	Graph indexation	1 graph	75m19	1.98	30.4
	Mapping reads	21,389,196 short read pairs	147m28	7	17.5
	JSON conversion	1 GAMP file	53m21	75	0.12
	JSON parsing	1 JSON file + 1 GFA file + 1 pickle file	110m44	0	5.7
	Abundance computing	1 Gene abundances table	0m4	0	0.68

Table S1. `StrainFLAIR` performances on simulated and mock datasets.

S1.5 Distance between the selected genomes in the simulated experiment

We estimated the distance between the complete genomes of the selected strains using fastANI (Average Nucleotide Identity). FastANI uses an alignment-free algorithm to estimate the average nucleotide identity between pairs of sequences.

	K-12	IAI39	O104:H4	Sakai	SE15	Santai	BL21-DE3	RM8426
K-12	100	97.0652	98.3769	97.8703	96.8716	98.0362	98.9365	98.3657
IAI39	97.037	100	96.9742	96.7417	97.1289	96.9295	97.0197	96.8987
O104:H4	98.3059	96.9521	100	97.4788	96.8007	97.8896	98.249	98.7212
Sakai	97.7497	96.8627	97.5094	100	96.6657	98.1523	97.7455	97.6125
SE15	96.8453	97.1064	96.9211	96.7362	100	96.7575	96.8141	96.7763
Santai	98.0073	97.0372	97.9584	98.1797	96.8199	100	97.9279	97.9077
BL21-DE3	98.9983	97.1721	98.4048	97.8227	96.8448	97.9616	100	98.3204
RM8426	98.306	96.9037	98.6801	97.5815	96.6907	97.8353	98.2567	100

Table S2. Distance between each pair of complete genome sequences from eight strains of *E. coli* as computed by fastANI.

All pairs showed a distance at least greater than 95%, highlighting the strong similarities between the strains. As a threshold, we although considered that beyond 99%, sequences were too similar to be considered and distinguished, additionally to the effect of sequencing errors. The fastANI results showed that none of the pairs exceeded this similarity threshold.

The strain *E. coli* BL21-DE3 was chosen as the unknown strain while the seven others would be used to build the reference variation graph. According to the results of fastANI, the strain BL21-DE3 closest genome in the present references is the strain K-12 with a similarity of 98.9%. Hence we expected to find evidences of the strain K-12 while analyzing a sample containing the unknown strain BL21-DE3.

S1.6 Detailed results from simulated datasets

#reads K-12	Method	O104:H4	IAI39	K-12	Sakai	SE15	Santai	RM8426
1,000	Expected	59.88	39.92	0.2	0	0	0	0
	`StrainFLAIR`	**56.47** (0.995)	**43.53** (0.989)	0 (0.309)	**0** (0.189)	**0** (0.151)	**0** (0.188)	**0** (0.212)
	`Kraken2`	38.91	60.72	**0.22**	0.04	0.07	0.03	0.02
5,000	Expected	59.41	39.6	0.99	0	0	0	0
	`StrainFLAIR`	**54.93** (0.995)	**42.46** (0.989)	2.6 (0.546)	**0** (0.202)	**0** (0.153)	**0** (0.2)	**0** (0.227)
	`Kraken2`	38.61	60.25	**0.99**	0.04	0.07	0.03	0.02
10,000	Expected	58.82	39.22	1.96	0	0	0	0
	`StrainFLAIR`	**54.12** (0.994)	**41.96** (0.989)	3.92 (0.709)	**0** (0.21)	**0** (0.155)	**0** (0.211)	**0** (0.234)
	`Kraken2`	38.26	59.69	**1.9**	0.04	0.07	0.03	0.02
25,000	Expected	57.14	38.1	4.76	0	0	0	0
	`StrainFLAIR`	**52.14** (0.994)	**40.58** (0.989)	7.27 (0.878)	**0** (0.208)	**0** (0.153)	**0** (0.215)	**0** (0.234)
	`Kraken2`	37.23	58.1	**4.51**	0.04	0.07	0.03	0.02
50,000	Expected	54.55	36.36	9.09	0	0	0	0
	`StrainFLAIR`	**49.25** (0.994)	**38.5** (0.989)	12.24 (0.949)	**0** (0.203)	**0** (0.15)	**0** (0.208)	**0** (0.23)
	`Kraken2`	35.63	55.6	**8.62**	0.04	0.07	0.03	0.02
100,000	Expected	50	33.33	16.67	0	0	0	0
	`StrainFLAIR`	**44.67** (0.994)	**35.04** (0.989)	20.29 (0.979)	**0** (0.202)	**0** (0.152)	**0** (0.207)	**0** (0.229)
	`Kraken2`	32.8	51.19	**15.85**	0.04	0.07	0.03	0.02
200,000	Expected	42.86	28.57	28.57	0	0	0	0
	`StrainFLAIR`	**38.12** (0.993)	**29.81** (0.988)	32.08 (0.99)	**0** (0.211)	**0** (0.159)	**0** (0.219)	**0** (0.237)
	`Kraken2`	28.31	44.18	**27.35**	0.04	0.08	0.03	0.02

Table S3. Reference strains relative abundances expected and computed by `StrainFLAIR` or `Kraken2` for each simulated experiment with variable coverage of the K-12 MG1655 strain. Best results are shown in bold. For `StrainFLAIR`, the proportion of specific genes detected is shown in parentheses.

Table S3 provides exhaustive results on simulated datasets when all queried strains are indexed in the variation graph. Table S4 provides exhaustive results on simulated datasets when one of the queried strain (BL21-DE3) is not indexed and highly similar to strain K-12.

#reads BL21-DE3	Method	O104:H4	IAI39	K-12	Sakai	SE15	Santai	RM8426
1,000	Expected	59.88	39.92	0.2*	0	0	0	0
	StrainFLAIR	**56.48** (0.995)	**43.52** (0.989)	0 (0.254)	**0** (0.189)	**0** (0.151)	**0** (0.192)	**0** (0.214)
	Kraken2	38.93	60.76	**0.11**	0.05	0.08	0.04	0.03
5,000	Expected	59.41	39.6	0.99*	0	0	0	0
	StrainFLAIR	**56.46** (0.995)	**43.54** (0.989)	0 (0.387)	**0** (0.216)	**0** (0.16)	**0** (0.218)	**0** (0.239)
	Kraken2	38.72	60.42	**0.5**	0.09	0.13	0.08	0.07
10,000	Expected	58.82	39.22	1.96*	0	0	0	0
	StrainFLAIR	**56.46** (0.995)	**43.54** (0.989)	0 (0.471)	**0** (0.236)	**0** (0.169)	**0** (0.243)	**0** (0.262)
	Kraken2	38.47	60.05	**0.92**	0.14	0.19	0.12	0.13
25,000	Expected	57.14	38.1	4.76*	0	0	0	0
	StrainFLAIR	**54.12** (0.995)	**41.72** (0.989)	**4.16** (0.584)	**0** (0.266)	**0** (0.177)	**0** (0.282)	**0** (0.298)
	Kraken2	37.75	58.93	2.16	0.28	0.34	0.25	0.29
50,000	Expected	54.55	36.36	9.09*	0	0	0	0
	StrainFLAIR	**52.77** (0.994)	**40.62** (0.989)	**6.61** (0.652)	**0** (0.284)	**0** (0.187)	**0** (0.307)	**0** (0.321)
	Kraken2	36.59	57.17	4.15	0.51	0.57	0.48	0.53
100,000	Expected	50	33.33	16.67*	0	0	0	0
	StrainFLAIR	**50.5** (0.993)	**38.63** (0.988)	**10.87** (0.687)	**0** (0.3)	**0** (0.196)	**0** (0.324)	**0** (0.338)
	Kraken2	34.53	54.03	7.68	0.91	0.98	0.91	0.96
200,000	Expected	42.86	28.57	28.57*	0	0	0	0
	StrainFLAIR	**46.96** (0.993)	**35.32** (0.988)	**17.72** (0.711)	**0** (0.318)	**0** (0.211)	**0** (0.346)	**0** (0.351)
	Kraken2	31.14	48.83	13.53	1.57	1.67	1.58	1.68

Table S4. Reference strains relative abundances expected and computed by `StrainFLAIR` or `Kraken2` for each simulated experiment with variable coverage of the BL21-DE3 strain, absent from the reference graph. BL21-DE3 being similar at 98.9% to K-12 strain (highest similarity compared to the other references), we expect that reads from BL21-DE3 will map this strain, hence its expected values are followed by an asterisk, as they correspond to BL21-DE3 strain abundances and not K-12. Best results are shown in bold. For `StrainFLAIR`, the proportion of specific genes detected is shown in parentheses.

S1.7 Detailed results for validation on mock datasets

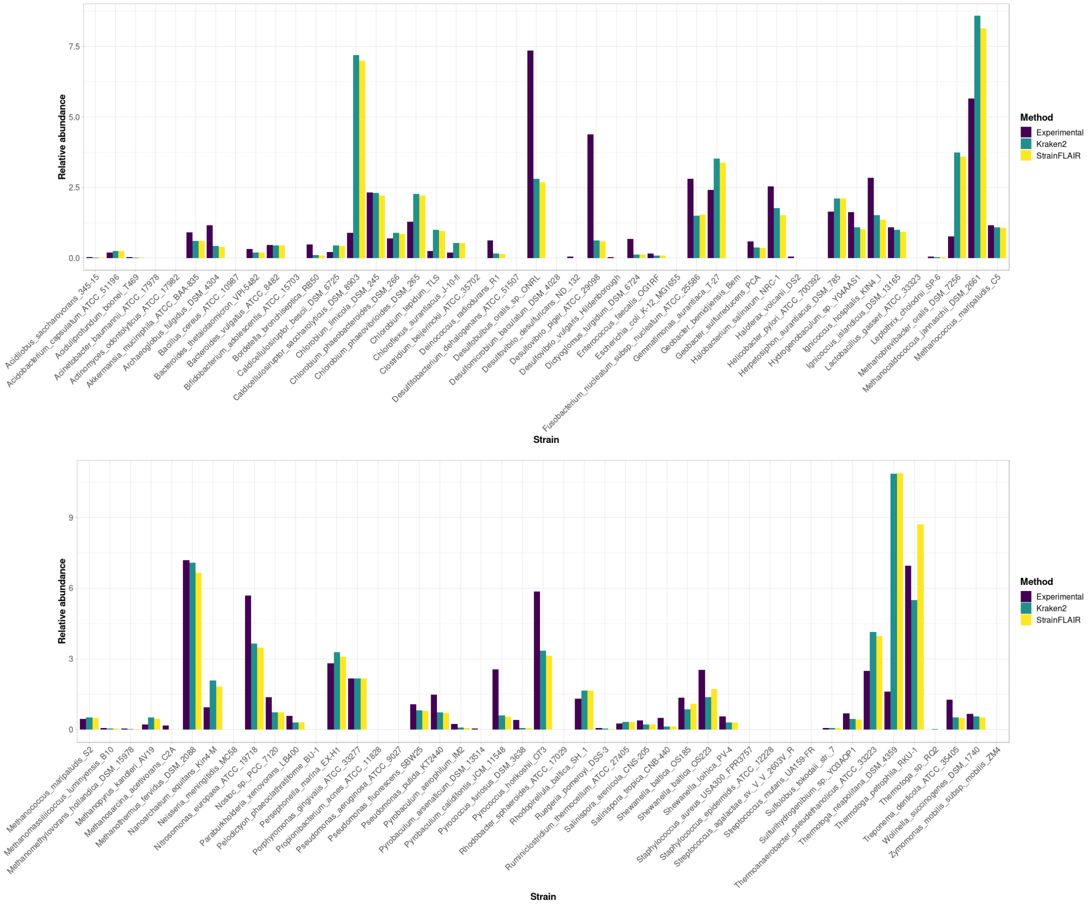

Figure S1. Experimental relative abundance compared to relative abundance computed by `StrainFLAIR` and `Kraken2`.

Figure S1 shows full results obtained on the mock dataset.

S1.8 Existing tools for strain identification and/or abundance estimation

Considering StrainFLAIR was designed to query a single sample, DESMAN was not suitable for this work as it needs multiple samples in order to compute variant co-occurrences.

Similarly, considering StrainFLAIR was designed to compute strain relative abundances, PanPhlan and StrainPhlan were not suitable as they do not provide such output.

StrainEst, DiTASiC, KrakenUniq and mixtureS had similar inputs and outputs compared to StrainFLAIR. Those tools were tested on two of the simulated datasets described in the main manuscript (mixtures of O104:H4 2011c-3493, IAI39, and K-12 MG1655 or BL21-DE3 at relative abundances of 50%, 33.33% and 16.67% respectively). It was enough to highlight their main differences with StrainFLAIR.

S1.8.1 StrainEst

Similarly to StrainFLAIR, StrainEst uses a set of reference genomes. *E. coli* K-12 MG1655 was used as the species reference needed in the StrainEst pipeline. It was also added for the clustering step of the representative genomes. The output is a relative abundance associated to each reference genome. Results are presented in Table S5.

While StrainEst gives slightly closer relative abundance estimations to the expected ones when the three strains from the mixture are represented in the references, it does not perform well with the mixture composed of an unknown strain (BL21-DE3). Aside from the relative abundances values that are farther than the ones provided by StrainFLAIR, the main issue is that StrainEst assigns an abundance to the strain RM8426 and not K-12 which is the closest strain to Bl21-DE3 and thus expected to capture the signal.

S1.8.2 DiTASiC

Similarly to StrainFLAIR, DiTASiC uses a set of reference genomes. The output is a table of read count estimates for each reference genomes associated with a standard error and p-value for those estimates. Read counts have been converted into relative abundances (percentages). Results are presented in Table S5.

While DiTASiC gives accurate relative abundance estimations when the three strains from the mixture are represented in the references, it does not perform well with the mixture composed of an unknown strain (BL21-DE3). Although in lower abundance than the three present strains in the sample, the absent strains are considered present even considering the p-values associated with the read count estimates, except for the strain SE15 (p-value = 0.55).

S1.8.3 KrakenUniq

KrakenUniq assesses the coverage of unique k-mers found in each species in a dataset. It has been used by building a custom database containing the same set of reference genomes as with StrainFLAIR. The output is a table of, among others, the average number of times each unique k-mer has been seen, and the coverage of the k-mers of the clade in the database, for each reference genome and their higher taxonomic levels. The number of times each unique k-mer has been seen has been converted into relative abundances (percentages). Results are presented in Table S5 with the coverage in parentheses.

Coverage values show a high discrimination between present and absent strains, with absent strains being in less than 0.1% in coverage. By using a threshold on this coverage, discarding the false-positive strains (Sakai, SE15, Santai and RM8426), the relative abundances computed are close to expected. However, IAI39 has a coverage of 0.5 while the two other present strains are at over 0.9, which could mislead the conclusion of IAI39 being the exact strain present in the sample, as it can been observed for the simulation with BL21-DE3 reads, the coverage associated with K-12 (0.34) is also higher than the absent strains and lower compared to present strains.

KrakenUniq was also used on the mock dataset and showed similar results compared to Kraken2 (sum of squared errors around 16 between KrakenUniq and Kraken2) except for two genomes which were drastically lower in abundance and close to abundances estimated by Kraken2 for absent strains. *Desulfovibrio piger* ATCC 29098 estimated abundance was around 1,000 times lower with KrakenUniq compared to Kraken2, and *Methanobrevibacter oralis* DSM 7256 around 60 times lower.

S1.8.4 mixtureS

mixtureS uses a single reference genome. The output is the inference of the number of haplotypes and an estimate of their relative abundance. Inferred haplotypes are not associated with known refer-

ences. For both simulated datasets, mixtureS gave similar results with 5 haplotypes predicted with abundances between 11 and 31% overall. Thus, those results could not be matched with the ones given by StrainFLAIR, StrainEst or DiTASiC, and consequently did not allowed accurate estimations in terms of number of strains in the mixtures nor in terms of abundances.

Mixture	Method	O104:H4	IAI39	K-12	Sakai	SE15	Santai	RM8426
with K-12	Expected	50	33.33	16.67	0	0	0	0
	StrainFLAIR	44.66	35.05	20.29	**0**	**0**	**0**	**0**
	StrainEst	48.64	32.97	18.39	**0**	**0**	**0**	**0**
	DiTASiC	**50.27**	**33.38**	**16.35**	**0**	**0**	**0**	**0**
	Kraken2	32.8	51.19	15.85	0.04	0.07	0.03	0.02
	KrakenUniq	38.27 (0.99)	26.14 (0.50)	15.28 (0.93)	5.08 (0.0017)	5.08 (0.0017)	5.08 (0.0017)	5.08 (0.0014)
with BL21	Expected	50	33.33	16.67*	0	0	0	0
	StrainFLAIR	**50.47**	38.64	**10.89**	**0**	**0**	**0**	**0**
	StrainEst	56.65	36.71	0	**0**	**0**	**0**	6.64
	DiTASiC	53.34	**34.72**	8.52	0.66	0.03	1.06	1.67
	Kraken2	34.53	54.03	7.68	0.91	0.98	0.91	0.96
	KrakenUniq	27.9 (0.99)	19.24 (0.50)	11.12 (0.34)	10.1 (0.02)	10.42 (0.02)	10.28 (0.03)	10.94 (0.04)

Table S5. Reference strains relative abundances expected and computed by StrainFLAIR or other tools for each simulated experiment. BL21-DE3 being similar at 98.9% to K-12 strain, we expect that reads from BL21-DE3 will map this strain, hence its expected value is followed by an asterisk, as it corresponds to BL21-DE3 strain abundance and not K-12. For KrakenUniq, additionally to the relative abundances computed from the average number of times each unique k-mer has been seen, the coverage value of the k-mers of the clade in the database was added in parenthesis. Best results are shown in bold.

REFERENCES

Paten, B., Eizenga, J. M., Rosen, Y. M., Novak, A. M., Garrison, E., and Hickey, G. (2018). Superbubbles, Ultrabubbles, and Cacti. In *Journal of Computational Biology*, volume 25, pages 649–663. Mary Ann Liebert Inc.
